# Bilateral hearing loss caused by anti‐NMDA receptor encephalitis with teratoma: A case report

**DOI:** 10.1002/ibra.12116

**Published:** 2023-06-25

**Authors:** Guo‐Fang Zhang, Tao Liang, Yi‐Kun Lv, Zhong Luo, Jun Zhang

**Affiliations:** ^1^ Department of Neurology Affiliated Hospital of Zunyi Medical University Zunyi China

**Keywords:** anti‐NMDA receptor encephalitis, autoimmune encephalitis, epilepsy, hearing loss, teratoma

## Abstract

Autoimmune encephalitis (AE) is an autoimmune disease in the central nervous system. Clinical manifestations include cognitive dysfunction, psychiatric‐behavioral abnormalities, epilepsy, motor disorders, speech disorders, and memory impairment. Some patients do not have the characteristic clinical manifestations of the disease when they see a doctor, so they are easily diagnosed incorrectly. Autoimmune antibodies originate from genetic and acquired factors. Clinical data have found a correlation between ovarian teratoma and autoimmune encephalitis. This case reports a 34‐year‐old woman who was diagnosed with teratoma‐associated anti‐N‐methyl‐D‐ aspartate receptor‐mediated autoimmune encephalitis called anti‐N‐methyl‐D‐aspartate receptor encephalitis with bilateral hearing loss in 2021. Through this case report, clinicians will pay attention to autoimmune encephalitis and raise awareness of the specific clinical manifestations of autoimmune encephalitis, and focus on early identification. It means that clinicians should be familiar with the representative clinical manifestations of the disease.

## Graphical abstract

We report a case of teratoma‐associated anti‐NMDA receptor encephalitis with bilateral hearing loss, which was diagnosed in our hospital, with symptoms of unilateral limb weakness and loss of consciousness, but hearing impairment was especially obvious during the course of the disease. The patient's symptoms improved significantly after the resection of the tumor. In this paper, the correlation between teratoma and autoimmune encephalitis was discussed, the pathogenesis of hearing impairment in teratoma‐associated autoimmune encephalitis was investigated, and the pathogenesis and treatment options of the disease were further discussed.

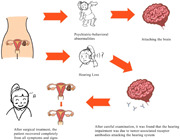

## 1 INTRODUCTION

Autoimmune encephalitis (AE) is an acute or subacute disease caused by immune responses to central nervous system (CNS) antigens.^1^, ^2^ Anti‐N‐methyl‐D‐aspartate (anti‐NMDA) encephalitis is an AE mediated by the production of anti‐NMDA receptor antibodies by B cells in the CNS, which affects neuronal calcium in‐flow and synaptic currents mainly through IgG (especially IgG1 and IgG3) binding to the NMDA receptor (NR1) subunit, leading to brain cell dysfunction. The underlying mechanism of antibody formation is unknown and is connected with ovarian teratomas in some individuals, primarily in women aged 25–35 years. In the 19th century, it was documented that patients' anxiety, fear, and dyskinesia were improved after ovarian teratoma resection.^3^, ^4^ Both tumors and infections can trigger anti‐NMDA encephalitis.^5^ It is believed that the neural tissue in the tumor stimulates the body to produce anti‐NMDAR antibodies. These antibodies bind to the neuronal NMDAR in the brain to induce AE, resulting in a series of symptoms, and immunotherapy and surgical removal of the tumor could improve the symptoms.^6^ Associated AE combined with tumors and hearing loss has been reported (Table 1).

**Table 1:** Autoimmune encephalitis combined with tumors and hearing loss.

Disease	Hearing loss	Combined tumors
Anti‐NMDA receptor encephalitis^7^	√	√
Kelch‐like Protein‐11 Encephalitis^8^, ^9^, ^10^	√	√
Pneumococcal meningitis^11^	√	×
Ma2 antibody encephalitis or brainstem encephalitis^9^, ^12^	√	√
aseptic meningitis and encephalitis^13^	√	√
MOG‐IgG‐associated brainstem encephalitis^14^	√	√
Other autoimmune encephalitides^7^, ^9^, ^14^	√	—

*Note*: Data screening and collection range for articles searchable in Pubmed from 2016 to 2021.

An increasing number of patients with ovarian teratoma combined with anti‐NMDAR encephalitis have been found clinically. Table 1 shows that AE has symptoms of hearing loss, but so far it has been reported relatively little, and the associated mechanisms have not been described. The improvement or even elimination of symptoms in patients after tumor resection provides some insight into the mechanisms involved. We report a case of teratoma‐associated anti‐NMDAR encephalitis with hearing impairment, in which clinical symptoms, especially hearing, improved markedly after tumor resection, which might be useful for clinicians treating AE.

## 2 CASE PRESENTATION

**Figure 1:**
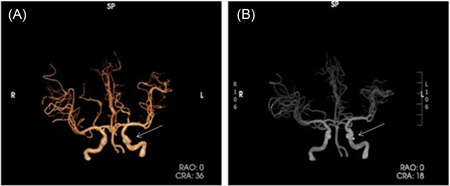
Computed tomography (CT) angiography imaging and revascularization. The patient's CT angiography imaging (A, arrow) and revascularization (B, arrow) of the carotid arteries showed a few calcified spots in the wall of the siphon segment of the left internal carotid artery, and no significant lumen stenosis was observed. [Color figure can be viewed at wileyonlinelibrary.com]

A 34‐year‐old female patient was first admitted to the Affiliated Hospital of Zunyi Medical University for “right limb weakness for 10 days and recurrent episodes of loss of consciousness for 7 h” on May 13, 2021. More than 10 days ago, numbness of the right limb with instability in holding objects appeared without any obvious cause and was not taken seriously. The patient's symptoms gradually worsened, and she developed bilateral hearing loss on the 4th day before admission. A head computed tomography (CT) scan showed no abnormalities, and only acupuncture rehabilitation was performed, but the symptoms did not improve significantly. Seven hours before admission, the patient experienced a sudden loss of consciousness without apparent cause that was accompanied by limb twitching (right limb first), gazing upward with both eyes, clenching of the teeth, and mouth foaming. The symptoms lasted about 2 min before going away on their own, and the patient regained consciousness after about 10 min. On clinical examination, cardiopulmonary abdominal physical examination without exception. Neurological examination revealed unresponsiveness, impairment of memory, computational ability, and temporal and spatial orientation. The muscle strength of the right upper limb was grade four (resistant to resistance, but worse than normal), with no increase and decreased muscular tension, and pathological symptoms were positive. The patient's CT angiography imaging of the carotid arteries showed a few calcified spots in the wall of the siphon segment of the left internal carotid artery, and no significant lumen stenosis was observed (Figure 1).

**Figure 2:**
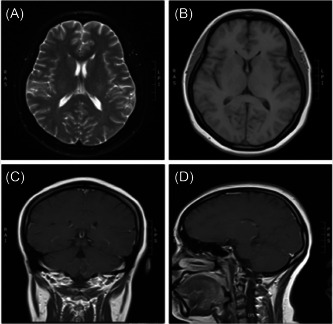
Cranial magnetic resonance imaging (MRI) examination. T2 FLAIR imaging (A), T1 imaging (B), T1 coronal (C), and T1 sagittal (D) showed no abnormal signals in the hippocampus and brain.

The patient's cranial magnetic resonance plain and enhanced scans, temporal bone CT, and CT angiography imaging of cerebral arteries showed no significant abnormalities (Figure 2).

## 3 DIAGNOSIS AND TREATMENT

**Figure 3:**
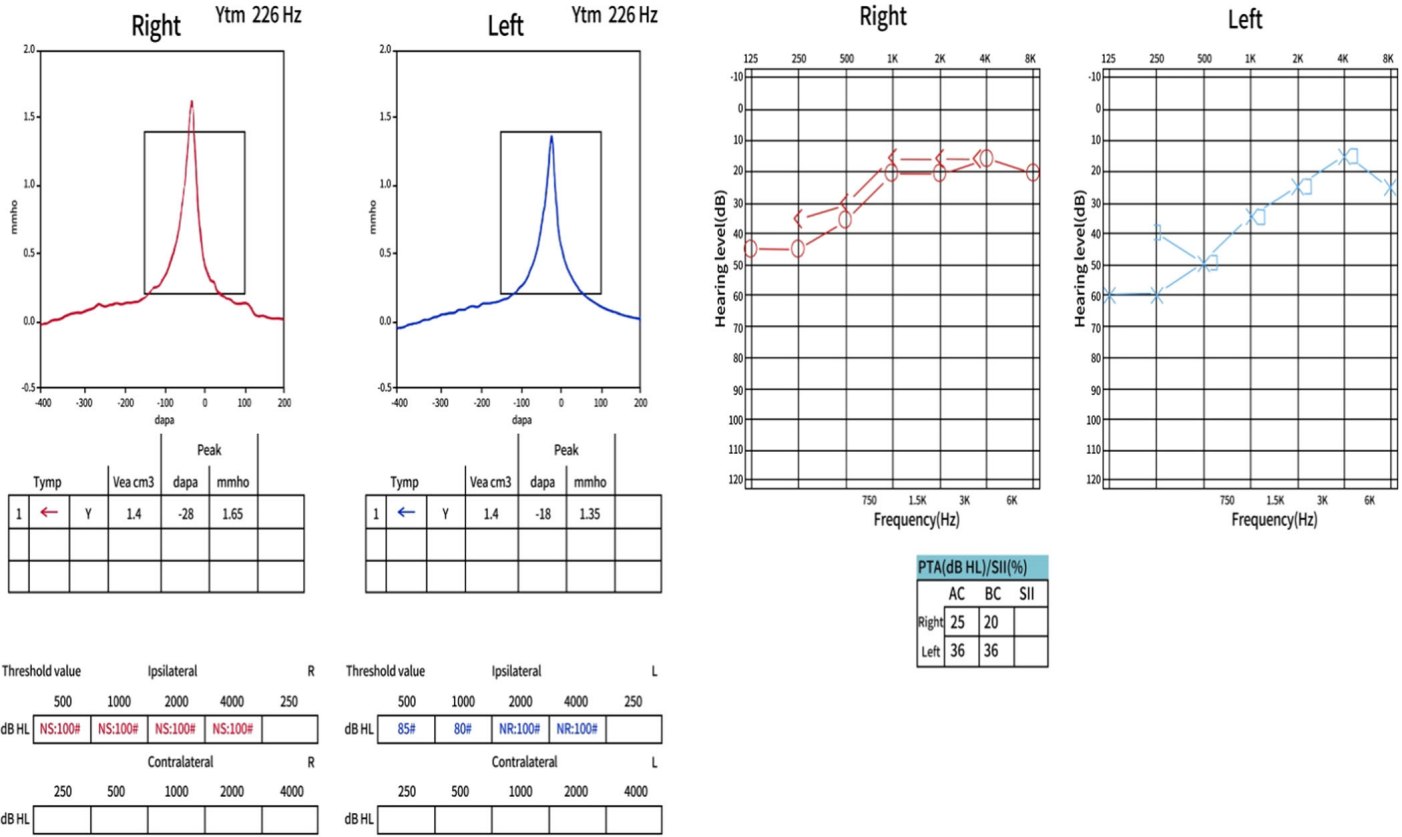
Acoustic conductance results (left) and the electroaudiometry (right). Acoustic conductance results show high peak amplitude in the right ear at 226 Hz examination with sound conduction greater than 1.5 mmho (left, arrow). Electroaudiometry shows greater than 25 dB at low frequencies in both the right and left ears, and the difference between air and bone conduction is less than 10 dB (right, arrow). [Color figure can be viewed at wileyonlinelibrary.com]

After electroencephalography and lumbar puncture (indirect immunofluorescence for serum and cerebrospinal fluid anti‐NMDAR antibody IgG, both at a titer of 1:10) in our hospital, the patient was diagnosed with “anti‐NMDAR AE” and was treated with hormonal suppression in our hospital. The patient then had progressive hearing loss with tinnitus, dizziness, headache, and discomfort. The patient's temporal bone CT did not show any abnormality. Rinne's test, Weber's test, and crude measurement of the patient's hearing did not show abnormality. However, the electroaudiometry and acoustic conductance maps showed severe bilateral sensorineural hearing loss, predominantly in the low‐frequency band (Figure 3), which were considered to have bilateral sensorineural deafness at the otolaryngology consultation, and vasodilatation and neurotrophic treatment were recommended, but the hearing symptoms did not improve.

**Figure 4:**
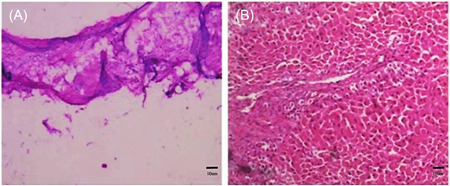
Paraffin and cryopreservation (grossly visible hairs, but no images left). Paraffin and cryopreservation results indicated a mature teratoma on the left adnexal mass (A) and a luteinizing cyst on the right ovary (B). [Color figure can be viewed at wileyonlinelibrary.com]

Because hormone shock therapy was ineffective, she was transferred to the intensive care unit for plasma exchange combined with high‐dose hormone therapy (methylprednisolone sodium succinate 1 g/day intravenous infusion, reduced to 500 mg/day on the 3rd day after admission to the intensive care unit and gradually reduced to 60 mg/day orally post surgery). However, her symptoms were poorly controlled after hormone shock therapy plus three plasma replacements, and the symptoms gradually worsened, with sporadic periods of mental clarity, indifferent expression, and little speech. The patient's gynecologic ultrasound showed a mixed echogenic mass in the left adnexa and a cervical cyst. After consultation with specialists from various departments, the possibility of teratoma in the left adnexal mass was considered, and AE was associated with the left adnexal mass. Thus, the left ovarian teratoma and the right ovarian corpus luteum cyst were removed laparoscopically by rapidly excluding contraindications to surgery. A post‐surgery pathological biopsy of the tissue indicated a grayish‐white cyst with a grayish‐white cut surface containing hairs and nodules. Paraffin and cryopreservation results indicated a mature teratoma on the left adnexal mass and a luteinizing cyst on the right ovary (Figure 4).

## 4 POSTOPERATIVE

The patient's symptoms gradually improved after surgery, and on the 25th day post surgery, the patient's symptoms and signs had completely disappeared, and she was discharged from the Department of Neurology. Based on the patient's clinical presentation, the post‐surgery symptoms had completely recovered, and a teratoma‐associated anti‐NMDAR encephalitis diagnosis was established. Nevertheless, in the 8th month after surgery, the patient was reexamined by lumbar puncture and serum, and cerebrospinal fluid anti‐NMDAR antibody IgG was detected by cell‐based assay, all with a titer of 1:30. Clinical details of the patient is shown in Table 2.

**Table 2:** Patient‐related clinical details.

Clinical stage	Therapeutic measures	Treatment results
Stage I	Hormone (methylprednisolone sodium succinate 1 g/day)	Ineffective
Stage II	Hormone (methylprednisolone sodium succinate 1 g/day) + Plasma exchange	Worse
Stage III	Surgery	Effective
Stage IV	‐	Full recovery on the 25th day post surgery

## 5 DISCUSSION

NMDAR is an ionotropic glutamate receptor that is widely distributed in the brain and is the basis of excitatory neurotransmitter transmission. In the CNS, glutamate binds to NMDAR to generate excitatory postsynaptic potentials to maintain normal brain activity and plays an influential role in CNS development, memory formation, synaptic function, and neuropsychiatric health.^15^, ^16^ When the body produces antibodies against NMDAR, it interferes with the binding of NMDA to glutamate and affects the brain's normal activity. Anti‐NMDAR encephalitis is a form of AE mediated by anti‐NMDAR antibodies. The functions of NMDAR include adjustment of synaptic transmission, remodeling, and participation in learning memory, and its dysfunction is associated with brain development, psycho‐behavioral abnormalities, and neurodegeneration; for the brain, glutamate release depends on binding to NMDAR to maintain the normal physiological electrical activity of the brain. NMDAR autoantibodies are mainly synthesized by peripheral or intrathecal immune cells, somatic cell mutations, ectopic tumor expression, or stimulated by antigen exposure after infection, and their target is the NMDAR NR1 subunit, which can cross the blood–brain barrier and cause synaptic dysfunction.^17^, ^18^, ^19^, ^20^

Related data show that about half of female AE patients have concomitant ovarian teratomas. Currently, tumor correlation, especially teratoma, is considered one of the etiologies of the disease.^21^ Teratoma‐associated anti‐NMDAR encephalitis has two mechanisms; first, the tumor releases antibodies into the bloodstream; second, tumor tissue exposure stimulates the body to produce antibodies; regardless of the source, all functional pathways involved in NMDAR are affected to varying degrees as anti‐NMDAR antibodies enter the brain.^15^, ^19^ According to current clinical data, hearing symptoms are less common in patients with teratoma‐associated anti‐NMDAR encephalitis, and Cheng et al. reported a case of ovarian teratoma‐associated anti‐NMDA receptor encephalitis with hearing symptoms as the first presentation and significant improvement in hearing after tumor resection and return to normal hearing after discharge from the hospital,^7^ consistent with our report. In 2014, a case of anti‐NMDAR encephalitis with hearing loss and cardiac arrhythmia in a man was reported. Still, no associated tumor was found in his body. The patient's symptoms did not improve particularly well under hormone and immunoglobulin treatment, and the mechanism of hearing loss in anti‐NMDAR encephalitis only stopped at the immune‐mediated levels from the treatment.^22^ The present case differs from the previous report that the patient's primary symptoms were motor impairment and psychiatric symptoms,^7^ and the secondary symptoms were hearing symptoms. After admission, motor symptoms did not progress or improve, psychiatric symptoms appeared intermittently, and hearing symptoms gradually deteriorated.

Therefore, the relationship between anti‐NMDAR encephalitis and hearing conduction pathways is thought‐provoking. Mechanisms of inner ear injury in autoimmune diseases include immune‐mediated vasculitis, microinfarction, neuritis, electrochemical disorders, direct immune attack, and associated drug toxicity.^23^, ^24^ Autoantibodies currently attacking the inner ear include anti‐Cogan peptide, anti‐linker protein 26, anti‐DEP1/CD148 and anti‐eutherovirus, anti‐neutrophil cytoplasmic antibodies, anti‐nuclear antibodies, rheumatoid factor and antiphospholipid, and anti‐heat shock protein hsp70.^25^, ^26^, ^27^ The pathogenic mechanisms of these antibodies are still under investigation, and teratoma‐associated anti‐NMDAR encephalitis hearing impairment is likewise poorly elucidated. NMADR is widely expressed in the brain and in varying degrees in the auditory nerve stem, cochlear inner hair cells, and neuronal synapses at all levels of the auditory transmission pathway.^28^, ^29^ The excitatory neurotransmitter of cochlear spiral ganglion neurons is glutamate, which plays an important role in hearing transmission through the receptor NMDAR.^30^ Most NMDARs are located on the axonal side of hair cells, distributed close to the nucleus, and hair cell ribbon synapses are the first afferent synaptic connections in the auditory pathway through which action potentials are transmitted to brain nerve fibers.^31^

NMDAR receptors are distributed in primary auditory afferent neurons, are involved in neurotransmission in the primary auditory and vestibular systems, and are present in almost all cells in the spiral and vestibular ganglia.^32^ The specificity of the distribution makes the inner ear highly susceptible to becoming an immune target in autoimmune encephalopathy, blocking the binding of glu to NMDAR, resulting in decreased synaptic currents and impaired auditory signal transmission, which is one of the reasons for the hearing loss in this patient. In addition to the corresponding psychiatric and motor deficits after admission, the progressive hearing loss in our patient indicates a progressive spread of anti‐NMDAR antibodies and further disease progression. From the perspective of immune‐related hearing impairment, hormonal shock, immunoglobulin, and plasma replacement are feasible treatment modalities. However, as the tumor grows and when the antibody titer in the body reaches a certain level, it is not easy to maintain an optimal balance between external clearance and antibody production. At this point, the patient's symptoms will further worsen, which is the reason for the progressive worsening of symptoms after admission to the hospital. In a previous study of 12 patients with teratoma‐associated anti‐NMDAR encephalitis and nine patients with tumor resection combined with chemotherapy, more than 80% of them showed significant improvement in their symptoms and returned to work, while antibody titers in blood and cerebrospinal fluid gradually decreased during follow‐up.^33^

In conclusion, AE should be considered when a mixed tumor is present in a patient who has poor healing following medication and immunotherapy. Early diagnosis of the tumor and timely surgical treatment should be completed, which can help patients recover.

## AUTHOR CONTRIBUTIONS

Guo‐Fang Zhang developed the idea for the study and wrote the paper. Tao Liang and Yi‐Kun Lv helped in writing the paper. Zhong Luo and Jun Zhang participated in the revision of the article. All authors contributed to the writing and revisions.

## CONFLICT OF INTEREST STATEMENT

The authors declare no conflict of interest.

## ETHICS STATEMENT

This case report was approved by the ethics committee of the Affiliated Hospital of Zunyi Medical University (Approval No: KLL‐2022‐766). Written informed consent was obtained from the patient to publish this case report.

## Data Availability

The data that support the findings of this study are openly available upon reasonable request.
